# Supplementary material for: Urine Proteome in Distinguishing Hepatic Steatosis in Patients with Metabolic-Associated Fatty Liver Disease
Source: Diagnostics (Basel). 2022 Jun 7;12(6):1412. doi: 10.3390/diagnostics12061412 (PMC9222194; doi:10.3390/diagnostics12061412)
Supplement: Supplementary file 1 [file diagnostics-12-01412-s001.zip › diagnostics- 1660342- Supplementary- new/Supplementary Table S1.pdf]

	p.normal.distribution	p.variance.homo	p.aov.test	p.adjust	Fold.Change
Q02487	0.1902747_0.1191746_0.576675	0.143674	0.001376	0.048991	1.137624
P62879	0.0297427_0.0005577_0.846832	0.133902	0.001332	0.048304	-0.78367
O00244	0.1714512_0.8945089_0.6413543	0.605714	0.001234	0.046051	1.583408
P02790	0.5217591_0.6556033_0.1389018	0.265493	0.00123	0.046051	1.064091
P21796	0.6871782_0.7665391_0.4449556	0.59298	0.001247	0.046051	0.671834
A0A087WSY6	0.9471132_0.9475427_0.2218103	0.167865	0.001127	0.045056	2.083767
P25325	0.1308141_0.7461507_0.2961592	0.221411	0.001123	0.045056	1.706102
Q04756	0.3451006_0.053453_0.1546685	0.401381	0.00113	0.045056	1.007656
Q16563	0.3819294_0.7044857_0.0735996	0.079339	0.001055	0.044767	0.643109
Q9H1U4	0.367563_0.1724319_0.6213971	0.374532	0.000975	0.042262	0.821607
Q96IU4	0.9512148_0.6482965_0.9340523	0.002255	0.000944	0.04185	2.145942
Q9P2S5	0.2713381_0.2807013_0.4452302	0.079226	0.000915	0.041473	1.571166
A0A0B4J1U7	0.2833599_0.3145945_0.9761419	0.434702	0.000777	0.037873	1.447061
Q13421	0.2885024_0.8602919_0.5439929	0.771852	0.000802	0.037873	1.509777
Q495M3	0.2418945_0.2323535_0.0529226	0.114836	0.000817	0.037873	-1.14764
Q96FV2	0.9042553_0.8701365_0.9036399	0.753485	0.000771	0.037873	1.512012
Q96KN2	0.373869_0.3630537_0.0332527	0.079606	0.000793	0.037873	2.009322
Q9Y6X5	0.9442482_0.5653044_0.5852769	0.938799	0.000704	0.037873	0.891012
Q6ZSJ9	0.1956797_0.1629616_0.9717383	0.350526	0.000647	0.035863	-1.03116
O43692	0.3411693_0.7609524_0.9089844	0.466842	0.000555	0.032009	-0.62648
P02763	0.0641181_0.5892774_0.522485	0.326017	0.000553	0.032009	2.896392
Q92484	0.5494176_0.272699_0.6554715	0.845958	0.000536	0.032009	0.648226
Q9NPG4	0.6247698_0.0561014_0.8441428	0.866124	0.000562	0.032009	1.586998
P62873	0.7813825_0.7293857_0.9344463	0.774366	0.000477	0.030709	-0.67448

P42785	0.2379034_0.1467743_0.3900858	0.143764	0.000419	0.027864	0.743865
Q9UGM3	0.0225304_0.159986_0.9885138	0.429452	0.000392	0.026954	-1.50135
Q96S37	0.4527949_0.0691785_0.1418663	0.4157	0.000366	0.026043	1.602168
P23515	0.5274043_0.7835544_0.5723543	0.242598	0.000328	0.025599	-0.61833
Q5FWE3	0.6494849_0.1007661_0.8468783	0.15732	0.000334	0.025599	-0.83958
O60262	0.0110977_0.0474427_0.9998874	0.887103	0.000287	0.024907	-1.47677
P14207	0.3057087_0.1670659_0.0334141	0.400524	0.0003	0.024907	2.007007
P57729	0.105554_0.5823161_0.0999582	0.881621	0.000294	0.024907	0.990004
Q9BY67	0.5198573_0.5595685_0.0158307	0.09972	0.000257	0.024362	1.27115
P04180	0.9440316_0.7625393_0.9992051	0.530013	0.000218	0.02169	0.938487
P20774	0.2657975_0.99419_0.6136665	0.098296	0.000208	0.02169	1.264643
P0DOY2	0.7531193_0.0574458_0.7987918	0.617661	0.000167	0.018516	1.323605
P00450	0.5568657_0.0332552_0.7989062	0.147027	0.000141	0.016543	2.158294
P22223	0.1749113_0.3288844_0.3366333	0.501672	0.000111	0.013862	1.59063
Q86YT9	0.0389313_0.2006133_0.7233405	0.234623	0.000111	0.013862	1.820303
P19320	0.9355928_0.6585284_0.9367027	0.499443	7.95E-05	0.011326	1.56428
P04217	0.3565663_0.9243591_0.7317905	0.662682	7.31E-05	0.011215	2.285819
P07359	0.4753214_0.9204787_0.1662074	0.823177	6.23E-05	0.011215	3.4246
P61020	0.9796118_0.675447_0.1823748	0.440245	6.99E-05	0.011215	-1.55847
P02750	0.7713604_0.7048973_0.1721405	0.57988	4.78E-05	0.009534	3.046082
P48551	0.136875_0.680566_0.539594	0.276137	4.48E-05	0.009534	2.632652
P10912	0.8603399_0.8885625_0.8665649	0.010729	2.21E-05	0.006233	2.775601
Q9NPH3	0.073882_0.1333099_0.4073511	0.939929	2.50E-05	0.006233	1.586451
Q96GW7	0.206434_0.0674284_0.7548009	0.402374	1.14E-05	0.00379	-1.56855
P01859	0.3641141_0.5427352_0.4696558	0.867741	4.40E-06	0.002234	1.402894

Q6UXB8	0.2153171_0.2398369_0.5994757	0.476469	4.80E-06	0.002234	1.500429
Q8WWZ8	0.5482773_0.2146166_0.0621498	0.932553	5.60E-06	0.002234	1.075225
P05543	0.1997206_0.5426221_0.6382469	0.192015	1.00E-06	0.001396	1.806856
Q9Y5Y7	0.1933701_0.9502393_0.2215046	0.238871	1.40E-06	0.001396	3.179743
