# Supplementary figures and images for: Urine Proteome in Distinguishing Hepatic Steatosis in Patients with Metabolic-Associated Fatty Liver Disease
Source: Diagnostics (Basel). 2022 Jun 7;12(6):1412. doi: 10.3390/diagnostics12061412 (PMC9222194; doi:10.3390/diagnostics12061412)

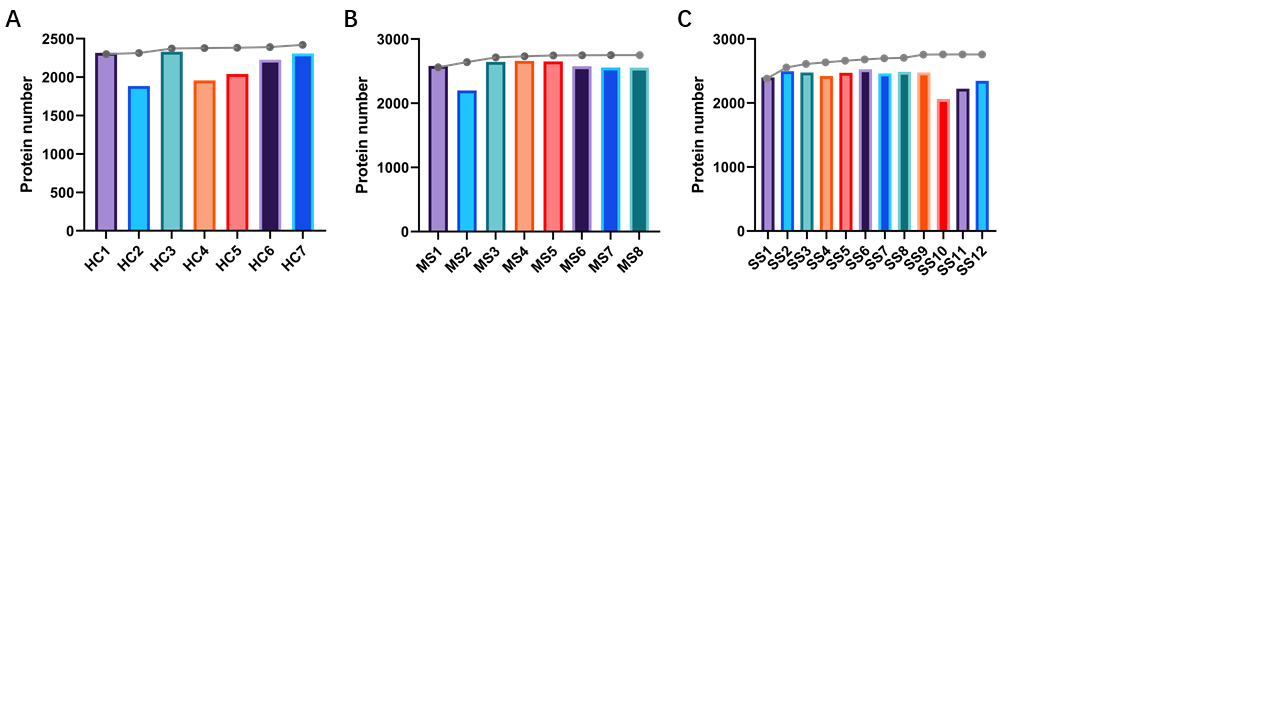

Supplement: Supplementary file 1 [file diagnostics-12-01412-s001.zip › diagnostics- 1660342- Supplementary- new/Supplementary Figure S1.tif]

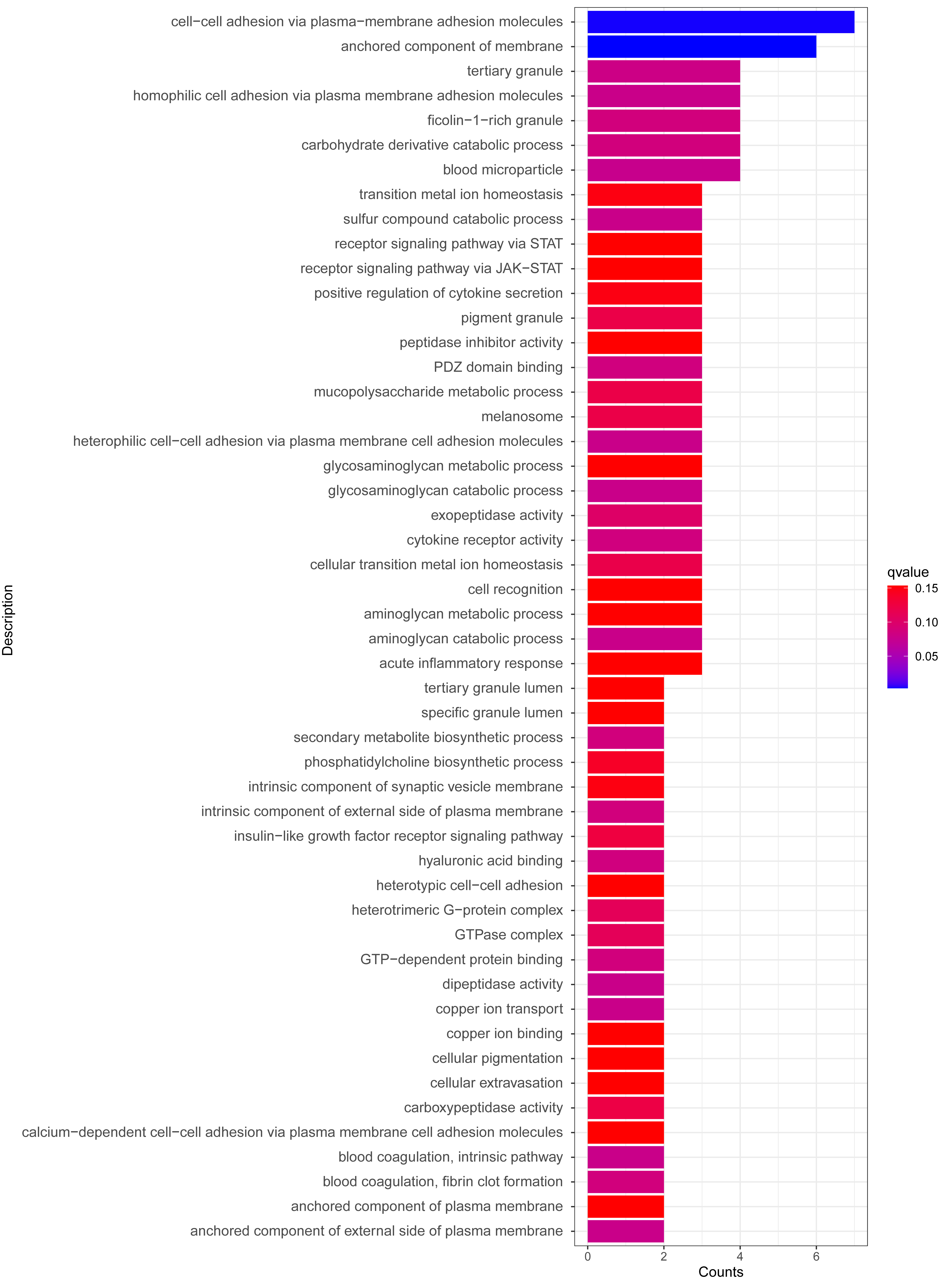

Supplement: Supplementary file 1 [file diagnostics-12-01412-s001.zip › diagnostics- 1660342- Supplementary- new/Supplementary Figure S2.tif]

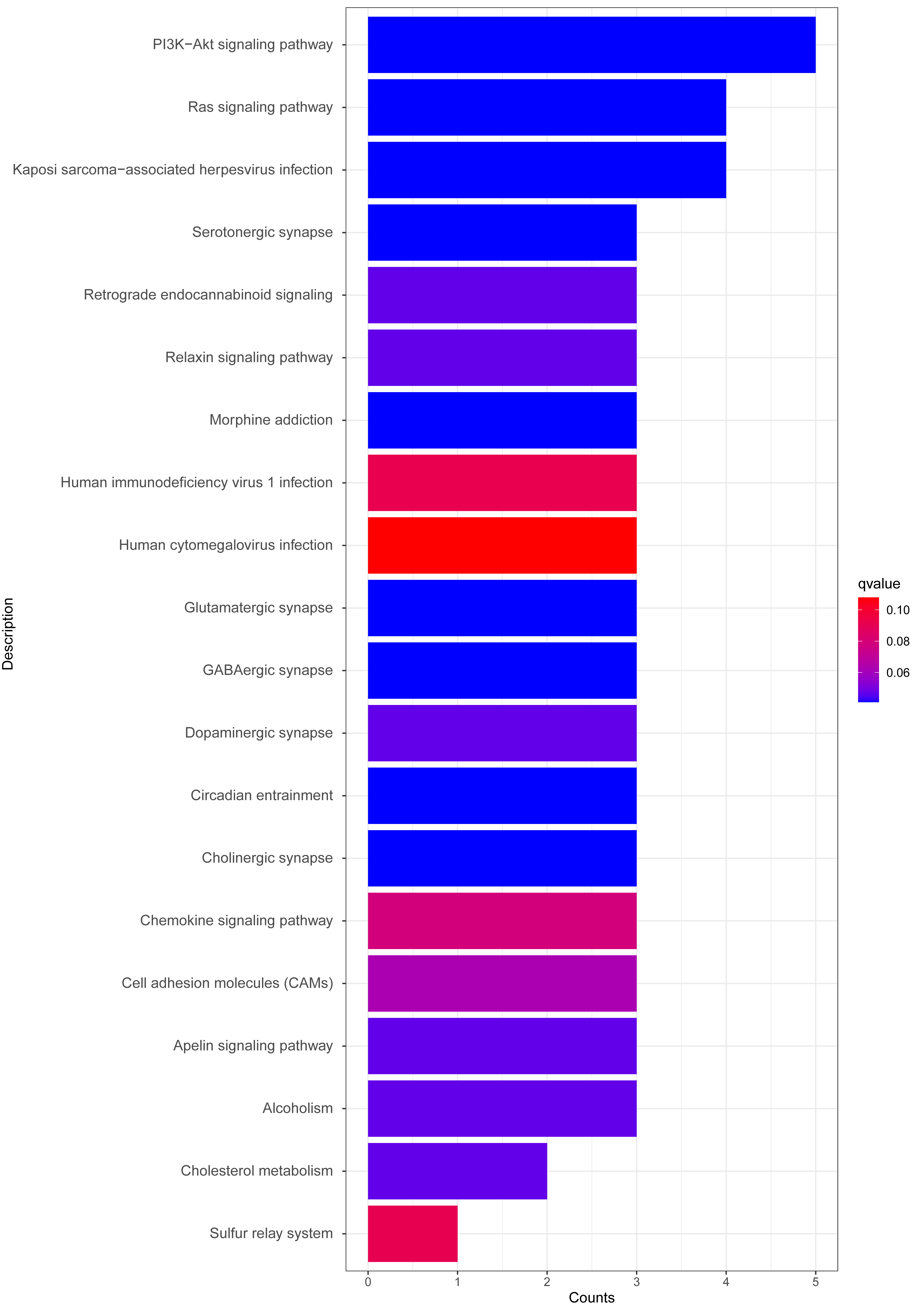

Supplement: Supplementary file 1 [file diagnostics-12-01412-s001.zip › diagnostics- 1660342- Supplementary- new/Supplementary Figure S3.tif]

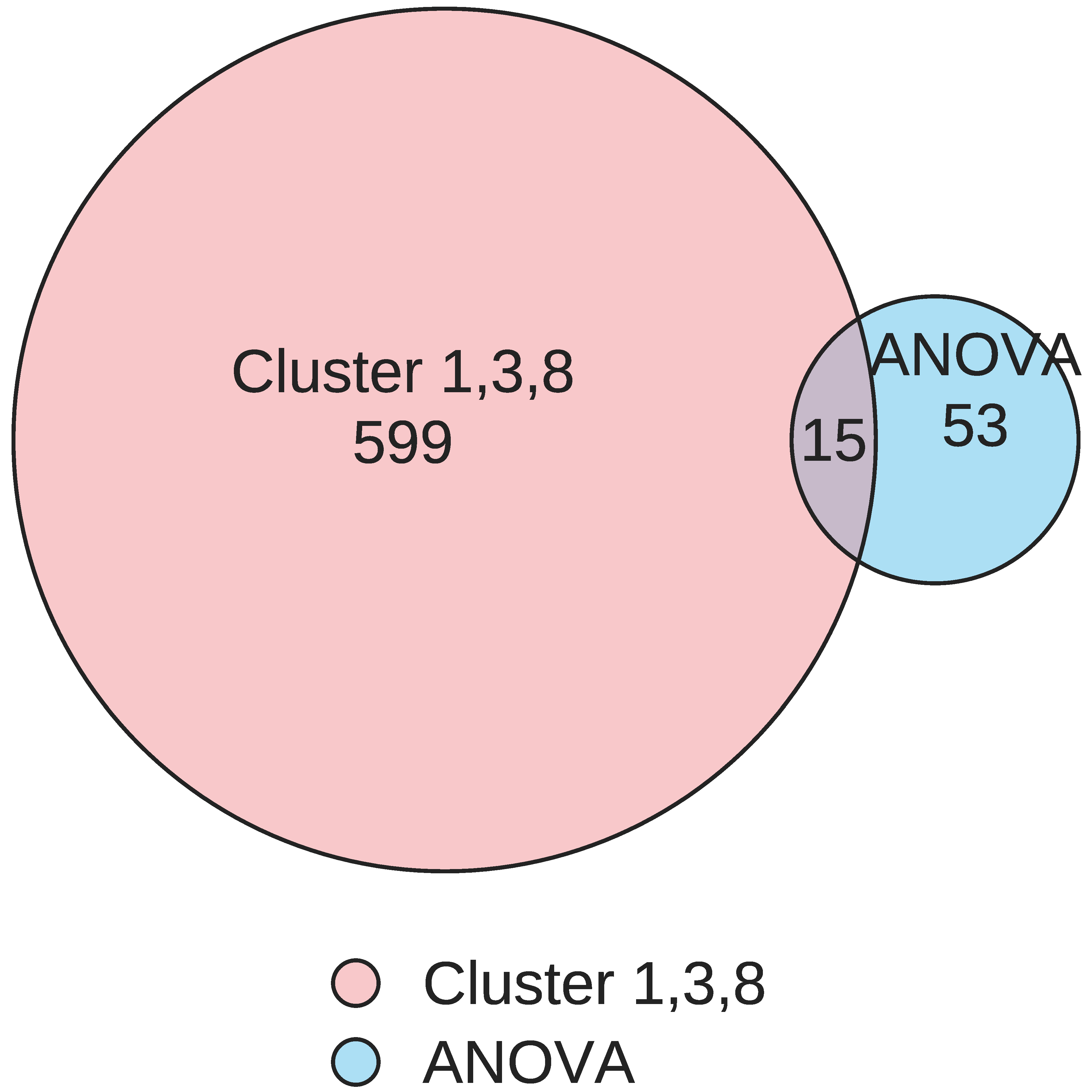

Supplement: Supplementary file 1 [file diagnostics-12-01412-s001.zip › diagnostics- 1660342- Supplementary- new/Supplmentary Figure S5.tiff]
